# Nicotinic Receptors in the Medial Habenula to Interpeduncular Nucleus Pathway: Modulators of Reward, Aversion and Emotion

**DOI:** 10.1111/ejn.70352

**Published:** 2025-12-11

**Authors:** Maria Ciscato, Mathilde Chouvaeff, Alexandre Mourot

**Affiliations:** ^1^ Brain Plasticity Unit CNRS, ESPCI Paris, PSL Research University Paris France

**Keywords:** acetylcholine, anxiety, avoidance behavior, cholinergic synapse, laterodorsal tegmentum, nicotine addiction

## Abstract

The medial habenula–interpeduncular nucleus pathway is a highly conserved and densely innervated brain circuit known for its unique cholinergic transmission and exceptional expression of nicotinic acetylcholine receptors. This pathway plays a critical role in regulating motivational and emotional processes, particularly those related to nicotine consumption, avoidance behaviors, and negative emotional states. Recent advances have revealed the intricate cellular architecture and receptor diversity of this system, highlighting how specific subunits of acetylcholine receptors influence both the rewarding and aversive properties of nicotine. Genetic and functional studies in rodents and humans point to this pathway as a key regulator of nicotine intake, with potential implications for addiction treatment. In this review, we examine the organization and molecular composition of nicotinic receptors within this pathway, describe their functional and behavioral roles, and explore how cholinergic signaling contributes to nicotine dependence, stress responses, and affective states.

## Graphical abstract

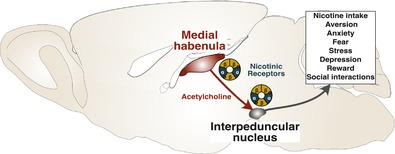

**Abbreviations**
18‐MC: 18‐Methoxycoronaridine
ACh: acetylcholine
AMPA: α‐amino‐3‐hydroxy‐5‐methyl‐4‐isoxazolepropionic acid
BAC: bed nucleus of the anterior commissure
CB1 receptor: cannabinoid receptor 1
ChAT: choline acetyl transferase
CHT1: choline transporter 1
CNS: central nervous system
CRF: corticotropin releasing factor
CRF1: corticotropin releasing factor receptor‐1
dlMHb: dorsolateral MHb
dMHb: dorsal MHb
dmMHb: dorsomedial MHb
DR: dorsal raphe
DTg: dorsal tegmental nucleus
FR: fasciculus retroflexus
GABA: γ‐amino butyric acid
GABAB receptor: GABA type B receptor
GLP‐1: glucagon‐like peptide‐1
GPR151: G protein‐coupled receptor 151
HCN: Hyperpolarization‐activated cyclic nucleotide–gated channels
HHIP: Hedgehog‐interacting protein
IPA: apical IPN
IPC: caudal IPN
IPDL: dorsolateral IPN
IPDM: dorsomedial IPN
IPI: intermediate IPN
IPL: lateral IPN
IPN: interpeduncular nucleus
IPR: rostral IPN
KI: knock‐in
KO: knock‐out
LDTg: laterodorsal tegmental nucleus
LHb: lateral habenula
MHb: medial habenula
MnR: median raphe nucleus
MOR: mu opioid receptor
MS: medial septum
nAChR: nicotinic acetylcholine receptor
NDB: nucleus of the diagonal band
NI: nucleus incertus
Nos1: nitric oxide synthase 1
SFi: septofimbrial nucleus
SST: somatostatin
TS: triangular septal nucleus
vcMHb: ventrocentral MHb
VGluT: vesicular glutamate transporters
vlMHb: ventrolateral MHb
vMHb: ventral MHb
vmMHb: ventromedial MHb
VTA: ventral tegmental area
WT: Wild‐type

## 1 Introduction

The medial habenula (MHb) and its primary downstream target, the interpeduncular nucleus (IPN), form a phylogenetically conserved pathway that plays a central role in regulating affective and motivational states (Viswanath et al. 2013; McLaughlin et al. 2017; Molas, DeGroot, et al. 2017; Ables et al. 2023). Situated between limbic forebrain regions and midbrain neuromodulatory centers, the MHb‐IPN axis is uniquely positioned to integrate emotional and contextual inputs and shape downstream neuromodulatory tone. Accordingly, this pathway has been implicated in a wide array of behaviors, including aversion, reward processing, stress responses, social interactions, and nicotine dependence (Fowler and Kenny 2014; Velasquez et al. 2014; Antolin‐Fontes et al. 2015; Molas, DeGroot, et al. 2017; Ables et al. 2023).

The MHb‐IPN circuit stands out in the central nervous system (CNS) for its exceptional density and diversity of nicotinic acetylcholine receptors (nAChRs) (Zoli et al. 2015). These pentameric ligand‐gated ion channels are critical for synaptic transmission and modulation within this pathway. Among the predominant subtypes expressed are heteromeric α3β4‐containing receptors, often in combination with accessory subunits such as α5. Strikingly, the genes encoding these subunits—*CHRNA5*, *CHRNA3*, *and CHRNB4*—are part of a gene cluster in the chromosome 15q25.1 region, a locus strongly associated with genetic risk for tobacco dependence and smoking‐related pathologies such as lung cancer (Mathis and Kenny 2019; Wills et al. 2022). Functionally, nicotinic modulation appears to play a key role in regulating the activity and output of the MHb‐IPN pathway, although the precise mechanisms and behavioral implications of this influence remain to be fully elucidated.

In this review, we focus on the nicotinic regulation of the MHb‐IPN pathway. We will detail the molecular composition and organization of nAChRs in this circuit, explore mechanisms by which cholinergic and nicotinic signaling influence MHb‐IPN function, and examine the behavioral and physiological consequences of such modulation. We will discuss emerging evidence linking this pathway to both the rewarding and aversive effects of nicotine, its broader roles in stress and emotional regulation, and its relevance to psychiatric and substance use disorders. Finally, we will highlight recent findings, including novel functional links to the ventral tegmental area (VTA), and identify key gaps in knowledge that remain to be addressed.

## 2 Anatomy of the MHb‐IPN Pathway

**FIGURE 1:**
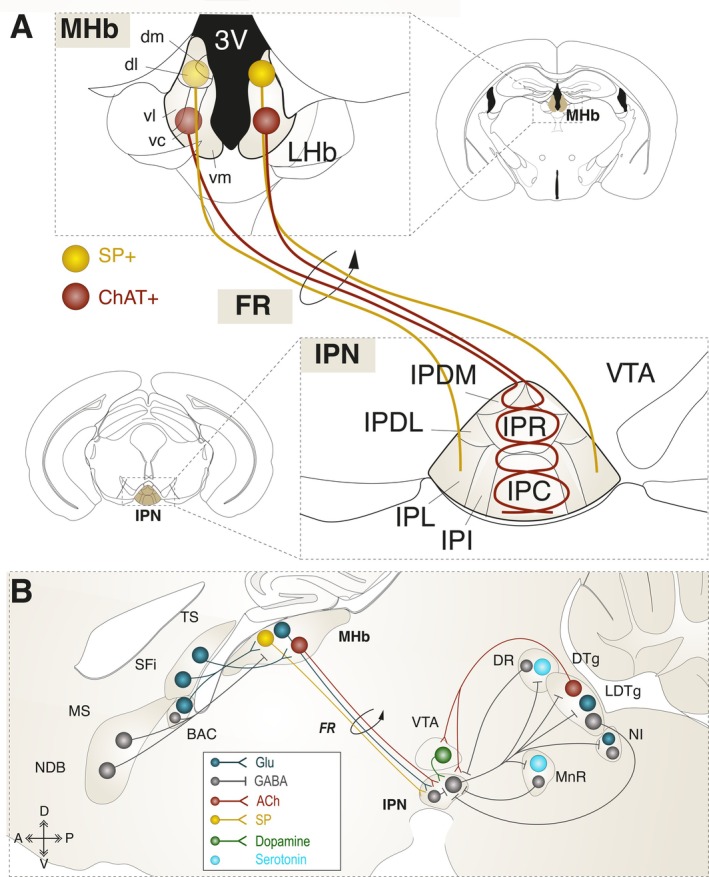
Anatomy of the MHb‐IPN pathway. (A) Different subnuclei of the MHb and IPN. The ventral MHb (vMHb) includes the ventrolateral (vlMHb), ventrocentral (vcMHb), and ventromedial (vmMHb; also called ventroinferior in some studies) subnuclei, while the dorsal MHb (dMHb) includes the dorsolateral (dlMHb) and dorsomedial (dmMHb) subnuclei. The IPN is subdivided into three unpaired subnuclei that lie along the midline and include the apical (IPA), rostral (IPR), and caudal (IPC) subnuclei, and four paired subnuclei that are found bilaterally and comprise the dorsomedial (IPDM), dorsolateral (IPDL), intermediate (IPI), and lateral (IPL) subnuclei. The IPA is more caudal and not shown here. dMHb neurons are mostly substance P positive and project to the lateral subnuclei (IPL and IPDL) of the IPN, whereas vMHb neurons are mostly cholinergic, and they project mostly on the medial (IPR and IPC) subnuclei of the IPN. (B) Major inputs of the MHb and outputs of the IPN. 3 V: third ventricle; BAC: bed nucleus of the anterior commissure; ChAT: choline acetyltransferase; dl: dorsolateral; dm: dorsomedial; DR: dorsal raphe nucleus; DTg: dorsal tegmental nucleus; FR: fasciculus retroflexus; IPC: central IPN; IPDL: dorsolateral IPN; IPDM: dorsomedial IPN; IPI: intermediate IPN; IPL: lateral IPN; IPN: interpeduncular nucleus; IPR: rostral IPN; LDTg: laterodorsal tegmental nucleus; LHb: lateral habenula; MHb: medial habenula; MnR: median raphe nucleus; MS: medial septum; NDB: nucleus of the diagonal band; NI: nucleus incertus; SFi: septofimbrial nucleus; SP: substance P; TS: triangular septal nucleus; vc: ventrocentral; vl: ventrolateral; vm: ventromedial; VTA: ventral tegmental area.

The habenula is a highly conserved epithalamic structure of the diencephalon located adjacent to the third ventricle which, in mammals, is subdivided into the MHb and lateral habenula (LHb) (Figure 1A) (Herkenham and Nauta 1977; Aizawa et al. 2012; Quina et al. 2017). The LHb has been extensively reviewed elsewhere recently (Mondoloni et al. 2022; Ables et al. 2023) and will not be covered here.

The MHb is subdivided into two dorsal (dMHb) and three ventral (vMHb) subnuclei (Figure 1A) (Aizawa et al. 2012; Wagner et al. 2014). Glutamatergic neurons are ubiquitous throughout the MHb, but the dMHb predominantly contains substance P–expressing neurons, whereas the vMHb is composed of cholinergic neurons (Ren et al. 2011; Aizawa et al. 2012; Hsu et al. 2014; Frahm et al. 2015). The posterior septum represents the main source of excitatory drive to the MHb (Herkenham and Nauta 1977; Qin and Luo 2009; Aizawa et al. 2012; Yamaguchi et al. 2013; Otsu et al. 2018) and also sends GABAergic projections (Figure 1B) (Herkenham and Nauta 1977; Qin and Luo 2009; Vickstrom et al. 2021). However, because of the atypical chloride gradient in MHb neurons, GABAergic transmission is functionally excitatory in this region (Kim and Chung 2007; Choi et al. 2016). Beyond septal inputs, the MHb receives more modest projections from subcortical regions, including the VTA, the locus coeruleus, and the raphe nuclei (Antolin‐Fontes et al. 2015; McLaughlin et al. 2017).

The IPN is an unpaired midline structure located in the ventral midbrain and subdivided into seven nuclei (Figure 1A) (Herkenham and Nauta 1977; Lenn and Hamill 1984; Antolin‐Fontes et al. 2015). It consists predominantly of GABAergic projection neurons and interneurons, with relatively sparse glutamatergic neurons that are localized mainly in the rostral (IPR), lateral (IPL), and caudal (IPC) parts of the IPN (Hsu et al. 2013; Lima et al. 2017; Quina et al. 2017). The IPN receives inputs from pontine nuclei, including the median (MnR) and dorsal (DR) raphe, the laterodorsal tegmental (LDTg), and the dorsal tegmental (DTg) nuclei (Lima et al. 2017; Quina et al. 2017) (Figure 1B). Additional inputs arise from several forebrain and midbrain regions, such as the septum, the medial preoptic area, the ventral thalamus, the central and ventral hypothalamus, the central gray, the periaqueductal gray, and the locus coeruleus (Lima et al. 2017; Quina et al. 2017). While several studies have reported functional dopaminergic inputs from the VTA (Zhao‐Shea et al. 2015; Molas, Zhao‐Shea, et al. 2017; DeGroot et al. 2020; Molas et al. 2022), the existence of a direct anatomical projection remains debated (Nasirova et al. 2021). Efferently, the IPN projects to two major neuromodulatory systems: the serotonergic raphe nucleus (MnR and DR) and the pontine cholinergic system via the LDTg (Figure 1B) (Hsu et al. 2013; Lima et al. 2017; Quina et al. 2017), and exerts inhibitory control over the nucleus incertus (NI), the central gray, and the medial part of the LHb (Lima et al. 2017; Quina et al. 2017).

The MHb is exceptional within the CNS in that it projects almost exclusively to a single target—the IPN—via the fasciculus retroflexus (FR), a phylogenetically conserved fiber bundle (Herkenham and Nauta 1977). These projections follow a highly precise topographical and neurochemical organization (Figure 1A): cholinergic neurons from the vMHb project bilaterally, with their axons crossing the midline multiple times at both the IPR and IPC levels (Quina et al. 2017), while substance P–expressing neurons from the dMHb project exclusively ipsilaterally and terminate primarily in the IPL (Molas, DeGroot, et al. 2017).

## 3 The MHb‐IPN Pathway Expresses a High Density and Diversity of Nicotinic Receptors

**FIGURE 2:**
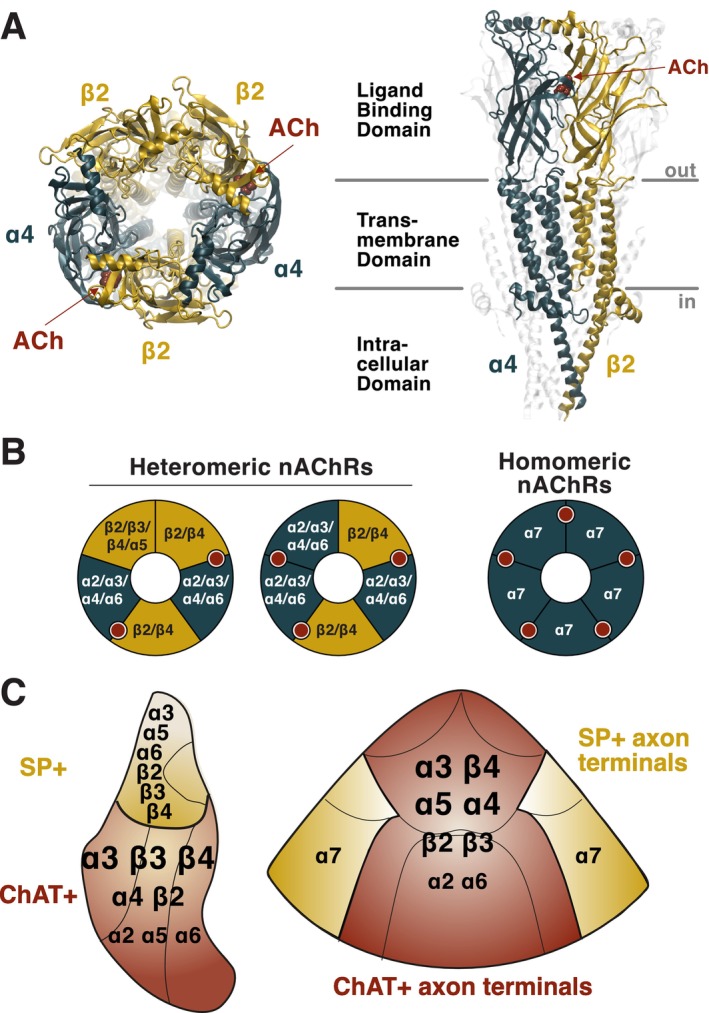
Structure, assembly, and expression patterns of neuronal nAChRs in the MHb‐IPN axis. (A) Various assemblies of α and β subunits into hetero‐ and homo‐pentameric structures with two, three, or five ACh binding pockets. (B) Structure of the two α, three β stoichiometry of human α4β2 nAChR in complex with ACh (PDB ID 8ST0 [Mazzaferro et al. 2024]). Left, top view; right, side view. (C) Patterns of expression of the different nAChR subunits in the MHb (left) and IPN (right). Font sizes indicate expression levels. Further details can be found in Table 1.

Neuronal nAChRs are ligand‐gated cation channels of the Cys‐loop receptor superfamily that are activated by the endogenous neurotransmitter ACh, as well as by nicotine, the addictive alkaloid found in tobacco (Taly et al. 2009; Nemecz et al. 2016; Gharpure et al. 2020). In the rodent CNS, 11 nAChR genes encode for eight α subunits (α2–α7, α9, α10) and three β subunits (β2–β4) (Gotti et al. 2006). These subunits assemble into pentameric structures surrounding a central pore that is permeable to sodium, potassium, and calcium ions (Figure 2A). Upon binding of agonists, the receptor undergoes a conformational change that opens the ion channel, allowing cation influx (Nemecz et al. 2016). Prolonged exposure to agonists leads to a transition into a high‐affinity, non‐conducting desensitized state (Taly et al. 2009; Nemecz et al. 2016).

Typically, α and β subunits co‐assemble to form heteromeric receptors, although some α subunits (e.g., α7 and α9) can form functional homopentamers (Taly et al. 2009). This combinatorial flexibility gives rise to a wide diversity of receptor subtypes (Figure 2B). Some combinations (e.g., α4β2, α4α6β2, α3β4, α3β4β3, α3β4α5 …) seem to preferentially occur in neurons, but the molecular determinants of preferential subunit assembly remain poorly understood (Gotti et al. 2006; Zoli et al. 2015). Subunit composition governs key pharmacological and biophysical features such as affinities for agonists, desensitization rates, and ion permeability (Fucile 2004; Gotti et al. 2006; Taly et al. 2009; Zoli et al. 2015). Affinity for ACh and nicotine is primarily governed by the α subunit (α4 > α3 > α7), and to a lesser extent by the β subunit (β2 > β4) (Gotti et al. 2006). Stoichiometry also matters: heteromers with three β subunits generally display higher agonist affinity than those with three α subunits (Moroni 2006). Desensitization kinetics differ across subtypes: β4* nAChRs desensitize less and recover faster than β2* receptors, while α7 nAChRs desensitize within milliseconds (Quick and Lester 2002; Gotti et al. 2009). Ion permeability depends on pore‐lining residues of all subunits: Homomeric α7 nAChRs exhibit exceptionally high calcium permeability, comparable to NMDA receptors (Seguela et al. 1993; Fucile 2004), whereas heteromeric α4β2 and α3β4 receptors have moderate calcium permeability, enhanced in receptors containing three alpha subunits (Tapia et al. 2007) or upon incorporation of α5 (Kuryatov et al. 2010).

The MHb‐IPN pathway is unique not only in its expression of a large variety of nAChR subunits, and notably rare ones such as α3 and β4 that are scarcely found elsewhere in the brain, but also in exhibiting one of the highest densities of nAChRs in the CNS (Gotti et al. 2009; Zoli et al. 2015). Table 1 and Figure 2C summarize current knowledge on the expression of nAChR subunits within the MHb and IPN. In the MHb, the highest nAChR expression is found in the vMHb and in the FR, with the α3, β3, and β4 being the most enriched subunits (Grady et al. 2009; Shih et al. 2014; Elayouby et al. 2021). The α7 subunit does not seem to be expressed in the MHb (Grady et al. 2009; Ables et al. 2017; Correa et al. 2019), but is present at low levels in a subset of GABAergic cells of the IPL (Jin and Drenan 2022). In the IPN, the α3 and β4 subunits are highly expressed, followed by α4, α5, β2, and β3. Consistent with their role in mediating cholinergic signaling, nAChRs are highly enriched in the IPR and IPC, which receive dense cholinergic innervation from the vMHb, and are expressed at lower levels in the lateral subnuclei (IPL, IPDL), where cholinergic input is sparse.

**TABLE 1:** nAChR subunit expression patterns in the MHb‐IPN pathway.

Subunit	MHb expression	IPN expression	Localization	Method	References
α2	No—low	Low—high	**MHb:** vMHb **IPN**: mostly IPR, but also found everywhere IPN	RNASeq RTqPCR ISH Single‐cell RT PCR IP	(Ishii et al. 2005; Grady et al. 2009; Scholze et al. 2012; Ables et al. 2017; Correa et al. 2019)
α3	High	Moderate—high	**MHb**: entire vMHb, lateral region of dMHb and FR CHT1 + neurons ChAT+ and ChAT‐ neurons Cytoplasm and extrasynaptic sites GPR151 + axons **IPN**: IPR VGluT2‐ neurons (i.e., GABAergic)	RNAseq RTqPCR ISH TRAP profiling Single‐cell RT PCR IP IHC Autoradiograms GFP‐KI mice PC + TABAC mice PC + SR16584	(Zoli et al. 1998; Sheffield et al. 2000; Salas et al. 2003; Salas et al. 2004; Grady et al. 2009; Quina et al. 2009; Frahm et al. 2011; Scholze et al. 2012; Shih et al. 2014; Ables et al. 2017; Correa et al. 2019; Tsuzuki et al. 2024)
α4	Moderate	Moderate—high	**MHb**: some areas vMHb (vlMHb) and FR ChAT+ and ChAT‐ neurons **IPN**:	RNAseq RTqPCR Single‐cell RT PCR IP GFP‐KI mice PC + Hypersensitive KI mice	(Sheffield et al. 2000; Fonck et al. 2009; Grady et al. 2009; Scholze et al. 2012; Shih et al. 2014, 2015; Ables et al. 2017; Correa et al. 2019)
α5	Low	Moderate—high	**MHb**: some areas vMHb (vlMHb and VcMHb) and some areas dMHb (dlMHb) **IPN**: IPR, IPC/IPI Projection neurons (to MnR, DTg/DRI) SST + neurons GABAergic neurons	ISH RNAseq TRAP profiling Single‐cell RT PCR IP GFP‐KI mice *CHRNA5* ^Cre^ mice PC + KO mice RTqPCR ^86^Rb^+^ efflux + KO mice shRNA knock‐down	(Sheffield et al. 2000; Salas et al. 2004; Grady et al. 2009; Fowler et al. 2011; Scholze et al. 2012; Hsu et al. 2013; Ables et al. 2017; Forget et al. 2018; Morton et al. 2018; Correa et al. 2019; Jin and Drenan 2022)
α6	Low	Low	**MHb**: some areas vMHb (vmMHb) and some areas dMHb (dmMHb) ChAT+ and ChAT‐ neurons **IPN**: IPR	RNAseq ISH Single‐cell RT PCR IP GFP‐KI mice PC + Hypersensitive KI mice PC + αCtxMII	(Le Novere et al. 1996; Sheffield et al. 2000; Grady et al. 2009; Scholze et al. 2012; Shih et al. 2014, 2015; Ables et al. 2017)
α7	No	Very low—low	**IPN**: mainly IPL GABAergic neurons	RNAseq RTqPCR Single‐cell RT PCR ISH PC + MLA/PNU‐120596	(Sheffield et al. 2000; Ables et al. 2017; Correa et al. 2019; Jin and Drenan 2022)
β2	Moderate	Moderate	**MHb**: entire vMHb and dMHb ChAT+ and ChAT‐ neurons **IPN**: mainly IPR	RTqPCR RNAseq Single‐cell RT PCR IP GFP‐KI mice ^86^Rb^+^ efflux	(Sheffield et al. 2000; Grady et al. 2009; Scholze et al. 2012; Shih et al. 2014; Ables et al. 2017; Correa et al. 2019)
β3	Moderate—high	Low—high	**MHb**: entire vMHb, some areas dMHb (dlMHb) and FR ChAT+ and ChAT‐ neurons **IPN**: mainly IPR	RNAseq RTqPCR Single‐cell RT PCR IP autoradiography GFP‐KI mice	(Sheffield et al. 2000; Cui et al. 2003; Grady et al. 2009; Shih et al. 2014; Ables et al. 2017; Correa et al. 2019)
β4	High	High	**MHb**: entire vMHb and dMHb, FR VGluT1 + CHT1 + neurons Neuropil region Extrasynaptic **IPN**: IPR VGluT2‐negative neurons (i.e., GABAergic)	RNAseq RTqPCR ISH IHC Single‐cell RT PCR TRAP profiling IP Autoradiograms GFP‐KI mice PC + β4E61C KI mice PC + KO mice PC + SR16584 [^3^H] ACh release + KO mice	(Zoli et al. 1998; Sheffield et al. 2000; Salas et al. 2003; Salas et al. 2004; Salas et al. 2004; Grady et al. 2009; Quina et al. 2009; Scholze et al. 2012; Beiranvand et al. 2014; Shih et al. 2014; Ables et al. 2017; Mondoloni et al. 2023; Tsuzuki et al. 2024; Jehl et al. 2025)

Abbreviations: αCtxMII: alpha‐Conotoxin MII (α3β2* and β3* nAChR antagonist); IHC: immunohistochemistry; IP: immunoprecipitation; ISH: in situ hybridization; MLA: Methyllycaconitine (selective α7 antagonist); PC: patch‐clamp electrophysiology; PNU‐120596: positive allosteric modulator of α7 nAChRs; RNAseq: RNA sequencing; RT PCR: reverse transcriptase PCR; scRNA‐seq: Single‐cell RNA sequencing; SR16584: specific α3β4 antagonist; TABAC: transgenic α3β4α5 cluster; TRAP: Translating Ribosome Affinity Purification.

The MHb and IPN display substantial cellular heterogeneity, with emerging transcriptomic and proteomic studies beginning to map nAChR expression to distinct cell populations (Hashikawa et al. 2020; Wallace et al. 2020; García‐Guillén et al. 2021; Sylwestrak et al. 2022). For instance, *CHRNA3* is often co‐expressed with G protein‐coupled receptor 151 (GPR151) and *CHRNB4* with choline transporter 1 (CHT1) in cholinergic MHb neurons, while *CHRNA5* is enriched in subsets co‐expressing somatostatin (SST) in the IPN, although precise cell‐type resolution of nAChR subunit distribution remains incomplete (Ables et al. 2017, 2023; Duncan et al. 2019; Caligiuri et al. 2022). Further research is needed to fully delineate these relationships and their functional significance.

## 4 Nicotinic Transmission Along the MHb‐IPN Pathway

### 4.1 The Source of ACh in the MHb Remains Elusive

Functional nAChRs—mostly α3β4*—are densely expressed in MHb cholinergic cells (Grady et al. 2009), within both somatic and dendritic compartments (Görlich et al. 2013; Shih et al. 2014; Banala et al. 2018; Morton et al. 2018; Passlick et al. 2018; Arvin et al. 2019; Elayouby et al. 2021; Jehl et al. 2025), strongly suggesting these neurons receive cholinergic innervation releasing ACh within the nucleus. Interestingly, MHb neuronal activity follows circadian rhythms, showing higher spontaneous activity during the light phase (Zhao and Rusak 2005), further hinting that ACh signaling might modulate MHb activity.

Despite this, the origin of cholinergic input to the MHb remains unknown, as tracing studies have failed to identify external cholinergic afferents (Chung et al. 2023). Septal inputs to the MHb were proposed to be a source of cholinergic innervation, yet these projections appear to act predominantly through muscarinic and not nicotinic receptors (Mu et al. 2022). A recent study showed that optogenetic stimulation of cholinergic neurons in the MHb increases the firing rate of neighboring, Channelrhodopsin2‐negative cholinergic neurons, an effect reduced in the presence of nAChR antagonists, suggesting a feedforward mechanism by which ACh facilitates local excitation through nAChRs (Chung et al. 2023).

Moreover, functional α3β4 nAChRs have been detected along the FR (Passlick et al. 2018; Tsuzuki et al. 2024), raising additional questions regarding the source of ACh that activates these receptors and their roles in these axons.

### 4.2 The MHb‐IPN Cholinergic Synapse

**FIGURE 3:**
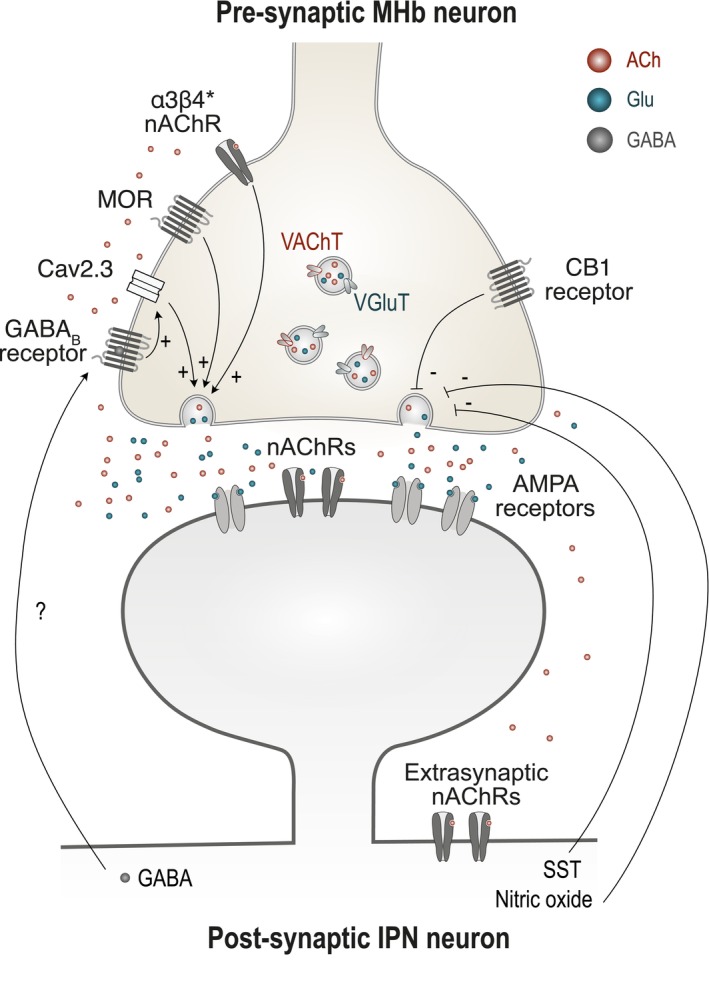
The MHb‐IPN cholinergic synapse. MHb cholinergic neurons co‐release ACh and glutamate onto IPN neurons. Vesicular transporters for both neurotransmitters (VAChT and VGluT) are co‐expressed on the same synaptic vesicles. nAChRs are present on MHb presynaptic terminals (α3β4* subtype), as well as on both synaptic and extrasynaptic sites on IPN neurons. MORs and GABA_B_ receptors facilitate neurotransmission, whereas CB1 receptors, SST, and nitric oxide act to suppress it.

It has long been known that ACh and nicotine potently activate IPN neurons and modulate presynaptic release of both glutamate and GABA, indicating that nAChRs are expressed both on postsynaptic IPN neurons as well as on MHb terminals and other afferents Mulle, Choquet, et al. 1991; Mulle, Vidal, et al. 1991; Léna et al. 1993; McGehee et al. 1995; Girod et al. 2000; Zhao‐Shea et al. 2013; Arvin et al. 2019; Mondoloni et al. 2023). VMHb cholinergic terminals coexpress vesicular transporters for both ACh and glutamate and corelease the two neurotransmitters (Figure 3) (Ren et al. 2011; Frahm et al. 2015). Two modes of neurotransmission have been proposed: fast, point‐to‐point synaptic signaling for glutamate, and slower, diffuse (volume) transmission for ACh. Indeed, brief optogenetic stimulation of MHb afferents evokes rapid AMPA receptor‐mediated responses, while sustained, high‐frequency stimulation is usually required to trigger slow inward currents mediated by nAChRs (Ren et al. 2011; Chittajallu et al. 2025). Given that ACh and glutamate are released from the same synaptic vesicles (Frahm et al. 2015), the slower time course of nicotinic currents suggests that nAChRs are predominantly extrasynaptic (Figure 3). However, other studies indicate that ACh can also mediate fast, time‐locked nicotinic postsynaptic currents in IPN neurons (Soria‐Gómez et al. 2015; Zhang et al. 2016), consistent with classical synaptic transmission. This apparent discrepancy may reflect the engagement of distinct IPN neuronal subtypes, or the recruitment of other signaling molecules (see Section 4.3), highlighting the need for cell‐type‐resolved investigations of cholinergic transmission within this pathway.

Presynaptic nAChRs can mediate cross‐transmitter modulation (Picciotto et al. 2012), as ACh is not only co‐released with glutamate but also enhances the release of both neurotransmitters (Girod et al. 2000; Girod and Role 2001; Frahm et al. 2015). Studies on IPN synaptosomes suggest that only the α3β4* and α3β3β4* nAChRs mediate ACh release (Grady et al. 2009), consistent with their enrichment in MHb axon terminals (Tsuzuki et al. 2024). Given their slow desensitization and sustained activation, α3β4* nAChRs may act as autoreceptors engaging a positive feedback loop that amplifies MHb activity.

The MHb‐IPN synapse exhibits a distinctive architecture that may restrict ACh diffusion via specialized IPN dendrites (Parajuli et al. 2020). At the ultrastructural level, IPN dendrites form U‐shaped crests that tightly wrap around MHb axons without glial separation, forming paired, asymmetric en passant synapses on opposite sides of the same dendrite (Lenn and Hamill 1984; Parajuli et al. 2020). A single MHb axon can form multiple contacts, creating a repetitive, localized excitatory pattern. This morphology may enhance synaptic efficacy by expanding the contact area and locally concentrating neurotransmitters within crest compartments.

### 4.3 Modulation and Plasticity of the MHb‐IPN Cholinergic Synapse

Cholinergic transmission at the MHb‐IPN synapse is dynamically tuned by several pre‐ and post‐synaptic modulators. For instance, blocking cannabinoid CB1 receptors in the vMHb increases ACh—but not glutamate—release in the medial IPN, indicating that endocannabinoids selectively inhibit cholinergic signaling (Figure 3) (Soria‐Gómez et al. 2015). Similarly, increased nitric oxide or SST levels suppress excitatory responses in the IPN following MHb terminal stimulation, suggesting strong presynaptic inhibition of ACh/glutamate release via SST receptors and cGMP‐dependent mechanisms (Ables et al. 2017).

Paradoxically, some classically inhibitory receptors promote excitation at this synapse. Activation of GABA_B_ receptors, which are typically Gi‐coupled and inhibitory, actually potentiates MHb‐IPN transmission by increasing presynaptic Ca^2+^ influx through R‐type voltage‐gated calcium channels (Cav2.3) and AMPA receptors (Zhang et al. 2016; Koppensteiner et al. 2024). This leads to enhanced co‐release of glutamate, ACh, and neurokinin B (Zhang et al. 2016), suggesting that GABA may act as a retrograde messenger to amplify excitatory drive. Notably, Cav2.3 channels localize presynaptically in MHb terminals (Figure 3), a rare feature compared to their typical postsynaptic localization elsewhere (Parajuli et al. 2020).

A similar paradox is observed with mu opioid receptors (MORs). Although MORs canonically inhibit neuronal activity, their activation at presynaptic cholinergic MHb terminals enhances synaptic output (Figure 3), particularly within the IPR (Chittajallu et al. 2025; Singhal et al. 2025). This is especially noteworthy given the abundant MOR expression in the MHb, one of the highest in the brain (Gardon et al. 2014). Importantly, nicotinic transmission becomes unmasked only when Kv1.2 potassium channels are inhibited, suggesting that voltage‐gated K^+^ channels normally suppress nAChR‐mediated responses under baseline conditions (Chittajallu et al. 2025). However, the physiological regulation of Kv1.2 activity remains unresolved.

Together, these mechanisms may underlie activity‐dependent plasticity at MHb–IPN synapses. High‐frequency stimulation of IPN neurons induces AMPA receptor–dependent GABA release, which retrogradely activates presynaptic GABA_B_ receptors, leading to long‐lasting facilitation of glutamate release (Koppensteiner et al. 2017). GABA_B_ receptor activation also triggers short‐term potentiation by shifting MHb synapses from tonic to phasic release, increasing the pool of readily releasable vesicles (Koppensteiner et al. 2024). Whether long‐term potentiation or depression occurs at these synapses—and whether nAChRs contribute to such plasticity—remains to be explored, particularly in the context of nicotine addiction and negative emotional state regulation.

## 5 Molecular and Cellular Effects of Nicotine on the MHb‐IPN Axis

### 5.1 Acute Effects

Nicotine increases the spontaneous firing of MHb neurons in slice preparations (Banala et al. 2018), an effect primarily mediated by α3β4* nAChRs (Görlich et al. 2013; Shih et al. 2014; Elayouby et al. 2021; Jehl et al. 2025) but also involving β2* (Elayouby et al. 2021) and α4* nAChRs (Shih et al. 2014) but not α5* nAChRs (Morton et al. 2018). Activation of α5* nAChRs by nicotine increases excitability indirectly, through the release of substance P and neurokinin B (Dao et al. 2014). Nicotine's effects on MHb firing are regionally specific: rapid, transient firing in vlMHb neurons; slower, sustained firing in vmMHb neurons; and no effect in the vcMHb (Shih et al. 2014). This heterogeneity likely reflects differences in nAChR subunit composition: fast‐desensitizing α4 predominates in the vlMHb, whereas α6 subunits are enriched in the vmMHb (Table 1) (Shih et al. 2015). Finally, nicotine also enhances glutamate release at MHb‐IPN terminals, a process regulated by presynaptic nAChRs, GPR151, and ACh co‐release (Girod and Role 2001; Frahm et al. 2015; Arvin et al. 2019; Antolin‐Fontes et al. 2020). Importantly, all of these findings come from slice recordings, where MHb inputs are severed. In vivo studies with calcium indicators have shown bulk activation of the MHb by nicotine (Caligiuri et al. 2022), while single unit electrophysiology recordings have revealed dose‐dependent effects: low nicotine doses activate MHb neurons, while higher doses suppress activity, likely through depolarization block mechanisms involving calcium‐activated chloride channels (Kawai et al. 2025).

In the IPN, neurons within the IPR also exhibit increased activity in response to acute nicotine application in brain slices, an effect mainly mediated by α5* (Morton et al. 2018) and β4* nAChRs (Mondoloni et al. 2023; Jehl et al. 2025). However, in vivo responses are more heterogeneous. Electrophysiological studies have identified two populations of IPN neurons: those activated and those inhibited by nicotine, intermixed within the IPR and IPC without strict regional segregation (Mondoloni et al. 2023; Jehl et al. 2025). The activation response is primarily mediated by β4* nAChRs, while the inhibition response likely involves other nAChR subtypes acting through yet‐undefined circuit mechanisms (Mondoloni et al. 2023; Jehl et al. 2025).

### 5.2 Chronic Effects

Nicotine intake perturbs endogenous cholinergic signaling due to its longer half‐life compared to ACh—approximately 2 h in humans and 6 min in mice (Matta et al. 2006). As a result, chronic nicotine promotes prolonged desensitization of nAChRs (Pidoplichko et al. 1997) and induces neuroadaptations that alter network excitability, including changes in nAChR expression levels (i.e., upregulation), subunit stoichiometry, and trafficking (Nashmi et al. 2007; Govind et al. 2009; Wills et al. 2022).

At the MHb level, chronic nicotine has dual effects: it reduces the acute response to nicotine, likely through nAChR desensitization (Dao et al. 2014), but simultaneously increases spontaneous firing, alters action potential waveforms, and enhances nicotine‐evoked glutamate release from MHb axon terminals (Arvin et al. 2019), likely through functional upregulation of nAChRs on the soma (Jin et al. 2020), dendrites (Banala et al. 2018; Jin et al. 2020), axons, and presynaptic terminals (Arvin et al. 2019). These changes result from an increase in receptor number rather than receptor potency (Banala et al. 2018), and may involve upregulation of α6* nAChRs (Henderson et al. 2014; Pang et al. 2016). Cholinergic neurons show no change in baseline or nicotine‐induced firing after chronic nicotine treatment (Görlich et al. 2013), but the effects may be region‐specific: vlMHb neurons, which predominantly express α4 (Shih et al. 2014), show no change in firing properties (Shih et al. 2015), consistent with α4 not being upregulated by nicotine (Nashmi et al. 2007). In contrast, vmMHb neurons, enriched in α6‐containing receptors (Shih et al. 2014), exhibit increased basal pacemaker firing but show a blunted acute nicotine response after chronic exposure (Shih et al. 2015).

At the IPN level, chronic nicotine induces nAChR upregulation in mice (Zhao‐Shea et al. 2013; Arvin et al. 2019) and rats (Tapia et al. 2022), but in a subtype‐ and cell‐specific manner. For example, the α7 subunit is selectively upregulated in SST‐negative cells while β3 and β4 subunits are upregulated in SST‐positive cells (Zhao‐Shea et al. 2013). Sex‐dependent differences have also been reported in rats: α7 upregulation predominates in males, while α5 increases in females (Correa et al. 2019). By contrast, α4* nAChRs are downregulated following chronic nicotine exposure (Nashmi et al. 2007). Functionally, these changes translate into potentiated nicotine responses in specific IPN neuron populations, particularly within the IPR (Banala et al. 2018; Arvin et al. 2019) and in SST‐positive neurons (Zhao‐Shea et al. 2013). However, opposite findings have also been reported: prolonged nicotine exposure can lead to downregulation and/or desensitization of β4* nAChRs in IPN neurons, resulting in reduced nicotine responsiveness both in brain slices and in vivo—an effect absent in β4 KO mice (Mondoloni et al. 2023). Discrepancies may stem from the recording context: persistent nicotine exposure in in vivo recordings maintains receptors in a desensitized state, whereas withdrawal conditions occurring in brain slices allow partial resensitization.

## 6 Behavioral Effects of Nicotine

A large body of human genetic studies has established a strong link between nicotinic signaling in the MHb‐IPN axis and nicotine dependence. Genome‐wide association studies have identified allelic variation in the *CHRNA5/A3/B4* gene cluster—encoding the α5/α3/β4 nAChR subunits that are highly enriched in this pathway—as a major risk for tobacco dependence and smoking‐related diseases (Berrettini et al. 2008; Bierut et al. 2008). In particular, the α5 D398N polymorphism, which reduces receptor function (Kuryatov et al. 2010), is associated with reduced aversive responses to nicotine (Jensen et al. 2015) and increased likelihood that light smokers progress to heavy smoking (Saccone et al. 2007; Bierut et al. 2008).

Furthermore, allelic variations in Hedgehog‐interacting protein (HHIP), an endogenous inhibitor of Hedgehog signaling, are associated with smoking‐related lung diseases. HHIP is enriched in MHb neurons where it modulates nAChR function through cholesterol‐dependent mechanisms and influences nicotine aversion (Caligiuri et al. 2022).

The MHb‐IPN axis also appears vulnerable to neurodegenerative effects of high‐dose nicotine, which can lead to degeneration of the FR (Carlson et al. 2000, 2001; Ciani et al. 2005), possibly through desensitization‐resistant α3β4* nAChRs. This raises the possibility that nicotine levels reached in habitual smokers might damage MHb axons, particularly those involved in nicotine aversion, potentially contributing to the transition toward compulsive tobacco use.

Finally, acute nicotine elevates blood glucose via a polysynaptic pathway from the MHb to the pancreas, while chronic nicotine increases circulating glucagon and insulin levels—mechanisms that may link cigarette smoking to an elevated risk of type 2 diabetes (Duncan et al. 2019). Together, these findings underscore the broad physiological and behavioral roles of the MHb–IPN pathway in nicotine addiction. In the following sections, we review rodent studies demonstrating that this pathway regulates both the aversive and rewarding properties of nicotine, thereby determining overall intake, and discuss its involvement in nicotine withdrawal and relapse.

### 6.1 Nicotine Aversion

In both humans and rodents, nicotine intake is thought to be titrated to maximize rewarding effects while avoiding aversive ones (Fowler and Kenny 2014; Wills et al. 2022). This balance is strongly dose‐dependent (Risinger and Oakes 1995; Matta et al. 2006; Liu et al. 2022): low doses preferentially activate VTA dopaminergic neurons via high‐affinity α4β2* nAChRs, often incorporating α6, promoting reinforcement (Tapper et al. 2004; Maskos et al. 2005; Pons et al. 2008; Tolu et al. 2013; Durand‐de Cuttoli et al. 2018; Nguyen et al. 2021). At higher doses, nicotine produces satiety and aversion, leading to reduced intake (Fowler et al. 2011; Frahm et al. 2011; Liu et al. 2022; Mondoloni et al. 2023). While aversion to nicotine can be mediated in part by VTA circuitry (Laviolette et al. 2002; Tolu et al. 2013; Liu et al. 2022; Wills et al. 2022), compelling evidence supports a central role for the MHb‐IPN pathway, particularly at high doses (Antolin‐Fontes et al. 2015; Wills et al. 2022).

Initial insights came from studies using *CHRNA5* knockout (α5 KO) mice, which display normal nicotine intake at low doses but consume significantly more at higher doses (Fowler et al. 2011; Bagdas et al. 2019). This excessive intake phenotype is reproduced by downregulating α5* nAChRs specifically in the MHb and reversed by region‐specific rescue of α5 expression (Fowler et al. 2011). Likewise, genetically modified rats carrying the α5 D398N polymorphism, which causes a partial loss of receptor function, exhibit greater nicotine self‐administration at high doses (Forget et al. 2018). Wild‐type (WT) mice typically regulate their intake, whereas α5 KO mice show a dose‐dependent escalation in consumption (Fowler et al. 2011), consistent with a reduction in aversive signaling (Fowler and Kenny 2014; Grieder et al. 2017). However, α5 is also expressed in the VTA, where its deletion diminishes the rewarding effect of nicotine as well. Specifically, VTA dopaminergic neurons in α5 KO mice fail to respond to low nicotine concentrations, preventing self‐administration at those doses (Morel et al. 2014). Thus, α5 deletion (or loss of function) has additive effects, dampening both the rewarding and aversive effects of nicotine, which disrupts titration mechanisms and promotes overconsumption.

**FIGURE 4:**
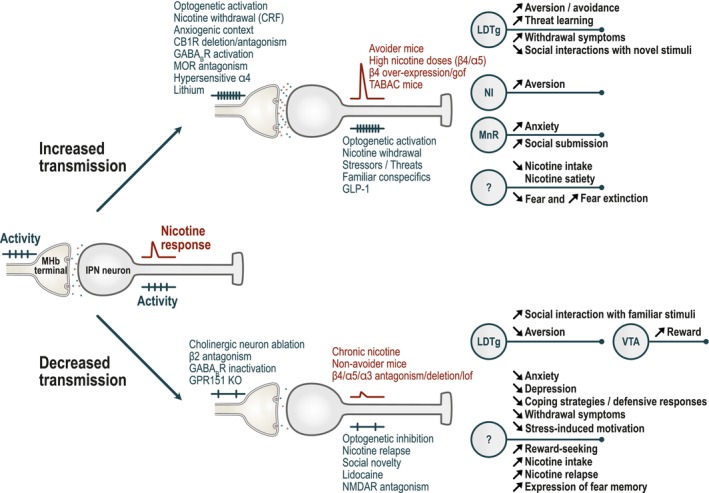
Receptors and circuits of the MHb‐IPN axis involved in nicotine effects, emotional regulation and social behaviors. Stimuli or conditions that affect neuronal excitability or synaptic transmission are shown in blue, whereas those influencing nicotine responses are in red. Overall, increased neurotransmission (top) promotes aversion, threat learning, anxiety, thereby suppressing nicotine intake. Conversely, decreased neurotransmission (bottom) relieves aversion and anxiety, alters coping strategies and enhances nicotine consumption. Question marks denote IPN outputs for which the functional target remains unknown. Lof: loss of function; gof: gain of function.

Complementary evidence indicates that the loss or reduction of α5, α3, or β4 nAChR subunits increases nicotine intake, while their overexpression intensifies aversion (Figure 4) (Wills et al. 2022). For example, TABAC mice, which overexpress β4 at endogenous sites, exhibit reduced intake and conditioned place aversion (Frahm et al. 2011; Husson et al. 2020). Similarly, local overexpression of β4 or β4 gain‐of‐function variants in the MHb induces aversion (Ślimak et al. 2014). Conversely, antagonizing IPN α3β4* nAChRs with 18‐Methoxycoronaridine (18‐MC) increases nicotine self‐administration (Glick et al. 2011). *CHRNB4* knock–out (β4 KO) mice show greater nicotine intake, particularly at high doses (Husson et al. 2020; Mondoloni et al. 2023), though some studies report divergent effects (Harrington et al. 2016). Local re‐expression of β4 in either the MHb or IPN restores normal intake (Husson et al. 2020; Mondoloni et al. 2023). Likewise, local knockdown of α3 or pharmacological blockade of IPN α3β4* nAChRs increases nicotine self‐administration (Elayouby et al. 2021).

Nicotine consumption varies markedly among individuals and is influenced by prior exposure (Fowler and Kenny 2014; Wills et al. 2022). Similar variability is observed in isogenic WT mice: roughly half avoid high nicotine concentrations, while others persist in consuming “aversive” doses (Mondoloni et al. 2023). Notably, nicotine intake correlates inversely with IPN neuron responsivity: animals with larger nicotine‐evoked responses in the IPN show stronger aversion, while those with blunted responses consume more. Accordingly, β4 KO mice exhibit drastically reduced IPN responses and universally lack aversion (Mondoloni et al. 2023).

More broadly, increased IPN activity drives aversion (Figure 4): optogenetic activation of IPN neurons induces place avoidance (Wolfman et al. 2018), and stimulation of α5‐expressing IPN neurons projecting to the raphe and tegmentum produces aversion, yet only after prior nicotine exposure (Morton et al. 2018), suggesting that exposure to nicotine experience triggers rapid adaptations in this pathway. Similarly, optogenetic activation of nucleus tractus solitarius neurons in the brainstem, which release glucagon‐like peptide‐1 (GLP‐1) onto IPN neurons, increases excitatory currents and elicits avoidance (Tuesta et al. 2017). Given that GLP‐1 neurons regulate satiety and inhibit food consumption (van Bloemendaal et al. 2014), and GLP‐1 receptors are enriched in α5‐positive IPN neurons, nicotine may engage these circuits to evoke satiety‐like signals that limit nicotine intake (Tuesta et al. 2017).

Conversely, inhibiting the IPN decreases aversion to the drug and promotes intake (Figure 4). α5 KO mice show attenuated IPN responses to nicotine (Morton et al. 2018), consistent with reduced aversive behavior (Fowler et al. 2011; Grieder et al. 2017). Local infusion of lidocaine (Fowler et al. 2011), or genetic deletion of the GPR151 receptor, which decreases MHb to IPN transmission (Antolin‐Fontes et al. 2020), raises the threshold for aversion and increases intake at high doses. On a circuit level, the LDTg has emerged as a downstream target: optogenetic silencing of IPN terminals in the LDTg blocks nicotine‐induced place aversion (Wolfman et al. 2018). Finally, chronic treatment with nicotine decreases IPN sensitivity to the drug, producing tolerance to its aversive effects (Mondoloni et al. 2023). This creates a vicious cycle in which reduced IPN sensitivity promotes escalating intake, reinforcing addiction—a process likely mediated by adaptive changes in nAChR function and circuit dynamics within the MHb–IPN axis.

### 6.2 Nicotine Reward

Emerging evidence indicates that the MHb‐IPN axis not only mediates nicotine aversion but also influences its motivational properties. For instance, silencing amigo1‐positive IPN neurons, which co‐express α5* nAChRs, SST, and Nos1, abolishes nicotine‐induced conditioned place preference (Ables et al. 2017). Similarly, selective knock down of ChAT in the MHb reduces glutamate co‐release in the IPN and eliminates nicotine preference (Frahm et al. 2015). These findings highlight that proper nicotinic transmission within the MHb–IPN circuit is required for nicotine to acquire reinforcing value.

Conversely, reduced activity in the IPN can alter the balance toward excessive reward, as shown using a chemogenetic method enabling local, pharmacologically specific, and sustained inhibition of β4* nAChRs. Blocking IPN β4* nAChRs enhances the dopaminergic response to nicotine in the VTA and induces preference for doses that are normally subthreshold for reward (Jehl et al. 2025), demonstrating that the IPN exerts inhibitory control over the dopaminergic reward system (Henderson 2025; Jehl et al. 2025). These findings align with previous studies showing that β4 subunit deletion (Harrington et al. 2016) or pharmacological blockade of α3β4* nAChRs in the MHb (McCallum et al. 2012) disrupt dopamine release in the nucleus accumbens. Notably, VTA dopaminergic neurons from β4 KO mice show elevated sensitivity to nicotine and altered conditioning (Harrington et al. 2016). At the circuit level, this IPN‐mediated brake involves projections to the LDTg (Jehl et al. 2025). Together, these findings suggest that nicotine reward is not solely a function of VTA dopaminergic activation, but is finely tuned by inhibitory signals originating in the IPN and relayed through the LDTg (Figure 4). This modulatory mechanism likely serves to balance nicotine's rewarding and aversive effects across different doses and contexts.

### 6.3 Nicotine Withdrawal and Relapse

The MHb‐IPN pathway is critically involved in both the emotional and somatic components of nicotine withdrawal. When nicotine levels drop, neuroadaptations induced by chronic exposure—such as nAChR desensitization, receptor upregulation, and network reorganization—unmask a state of cholinergic imbalance. This imbalance contributes to the emergence of somatic, affective, and motivational withdrawal symptoms that drive the urge to relapse. In rodents, nicotine withdrawal induces a broad spectrum of symptoms, including aversion, anhedonia, anxiety and fear‐like behavior, as well as somatic signs such as body and head shakes, paw tremors, scratching, grooming, and hyperalgesia (Koob and Moal 1997; Salas et al. 2009; Zhao‐Shea et al. 2013; Molas, DeGroot, et al. 2017; Wills et al. 2022).

Withdrawal can be precipitated either by nicotine cessation or systemic administration of non‐selective nAChR antagonists such as mecamylamine (Watkins et al. 2000). Notably, local microinjections of mecamylamine into the habenula or IPN—not into the cortex, VTA, or hippocampus—are sufficient to drive withdrawal, demonstrating the central role for the MHb‐IPN axis (Salas et al. 2009).

During chronic nicotine exposure, the IPI shows upregulation of corticotropin releasing factor receptor‐1 (CRF1), a key mediator of stress responses (Zhao‐Shea et al. 2015). Upon withdrawal, CRF released from the VTA activates IPI neurons through increased glutamatergic transmission from the MHb (Zhao‐Shea et al. 2015), contributing to aversion and anxiety during withdrawal (Figure 4) (Grieder et al. 2014). Sex differences are notable: females show stronger IPN activation during withdrawal, and CRF1 antagonism increases glutamate release only in females (Carcoba et al. 2022).

β4* nAChRs play a key role in the somatic manifestations of withdrawal: mice lacking β4 exhibit reduced withdrawal symptoms (Salas et al. 2004). Furthermore, MHb pacemaker cholinergic cells, which respond to nicotine via α3β4* nAChRs, become hypersensitive after withdrawal, and inhibition of these cells using HCN channel blockers precipitates somatic withdrawal symptoms (Görlich et al. 2013). At the IPN level, withdrawal is associated with increased activity of SST‐positive interneurons in the IPR, which upregulates β4* nAChRs during chronic nicotine exposure. Blockade of these receptors precipitates somatic withdrawal signs and induces c‐fos expression in SST‐negative projection neurons (Zhao‐Shea et al. 2013). These findings suggest that β4* nAChRs in SST‐positive interneurons act as a brake on withdrawal, and that their inhibition disinhibits projection neurons, driving somatic withdrawal behaviors.

Distinct nAChR subunits contribute differentially to withdrawal phenotypes. Both male and female rats show upregulation of IPN β3 and β4 subunits during nicotine withdrawal, while α2* and α3* nAChRs are selectively increased in males (Correa et al. 2019). The β4 subunit is primarily involved in somatic signs but not in affective components, as blocking α3β4 nAChRs in the MHb fails to alleviate withdrawal‐induced anxiety (Pang et al. 2016). In contrast, pharmacological blockade of α4β2* or α6β2* nAChRs in the MHb reduces anxiety‐like behavior in nicotine‐withdrawn mice (Pang et al. 2016). α5* nAChRs modulate withdrawal via neurokinin signaling: blocking neurokinin receptors in the MHb precipitates somatic withdrawal signs (Dao et al. 2014).

Overall, nicotine withdrawal increases the frequency of excitatory inputs and enhances IPN activity (Zhao‐Shea et al. 2013; Klenowski et al. 2022), particularly within the IPI (Zhao‐Shea et al. 2015). However, IPN firing transiently decreases during the expression of somatic withdrawal behaviors, possibly reflecting a coping mechanism to buffer aversive states (Klenowski et al. 2022). Optogenetic activation of IPN neurons precipitates both somatic withdrawal signs (Zhao‐Shea et al. 2013) and anxiety‐like behavior (Klenowski et al. 2022), whereas suppression of activity with optogenetics (Klenowski et al. 2022; Monical and Mcgehee 2025) or NMDA receptor antagonists (Zhao‐Shea et al. 2013) alleviates somatic withdrawal symptoms (Figure 4). At the circuit level, mecamylamine is thought to precipitate withdrawal symptoms by inhibiting GABAergic interneurons in the IPN, thereby disinhibiting projection neurons (Zhao‐Shea et al. 2013), which likely target the LDTg. Accordingly, during nicotine withdrawal, LDTg activity is suppressed and dopaminergic activity is decreased (Monical and Mcgehee 2025), supporting the existence of a shared IPN–LDTg–VTA pathway for both the positive (Jehl et al. 2025) and negative motivational aspects of nicotine dependence.

Beyond withdrawal, nicotinic signaling within the IPN also contributes to relapse‐like behaviors. KI rats carrying the human α5 D398N polymorphism exhibit enhanced nicotine‐induced reinstatement of nicotine‐seeking behavior (Forget et al. 2018). This relapse‐like behavior is associated with reduced neuronal activity in the IPN, and the degree of reinstatement negatively correlates with IPN responsivity (Figure 4). Brain slice recordings from these rats revealed a deficit in IPN neuron responses to nicotine, suggesting that blunted IPN activity facilitates relapse‐like behaviors and highlight a critical role for IPN α5* nAChRs in controlling relapse vulnerability.

## 7 Role of Endogenous Nicotinic Modulation in Negative Emotional States

The MHb‐IPN pathway plays a critical role in processing negative emotional states, such as stress, aversion, fear, and anxiety (McLaughlin et al. 2017; Molas, DeGroot, et al. 2017; Ables et al. 2023). This pathway integrates exteroceptive and interoceptive aversive cues, acting as a hub for modulating avoidance learning, defensive behavior, coping strategies, and emotional homeostasis. Notably, MHb neurons possess primary cilia (Foll and French 2018), which may enable them to sense cues from the cerebrospinal fluid and contribute to the integration of internal states. Although nAChRs within the MHb–IPN axis are known to mediate nicotine aversion and anxiety during withdrawal, the potential involvement of endogenous habenular ACh in modulating emotional processes remains largely underexplored; this possibility will be examined in the sections below.

### 7.1 Stress and Aversion

The MHb–IPN axis is robustly activated by stressful and aversive experiences such as foot shocks, tail lifts or physical restraints (Hashikawa et al. 2020; Klenowski et al. 2023; Liang et al. 2024; Williams et al. 2025). Optogenetic activation of MHb (Bailly et al. 2023) or IPN neurons (Wolfman et al. 2018), or blockade of MORs in the MHb with naloxone (Boulos et al. 2020), induces robust avoidance behavior (Figure 4). Importantly, visceral stressors such as lithium chloride injection also engages this pathway to drive aversion (Otsu et al. 2019), pointing to its sensitivity to interoceptive negative states. From a circuit point of view, the projections from the IPN to the NI were shown to amplify aversion (Liang et al. 2024), while those to the LDTg can directly elicit avoidance (Wolfman et al. 2018).

Several lines of evidence suggest that cholinergic signals within the MHb‐IPN axis regulate behavioral avoidance of negative stimuli. Nicotine itself produces aversion by activating nAChRs within this pathway (Wills et al. 2022), suggesting that endogenous ACh may exert similar effects. The finding that optogenetic activation of the pathway induces aversion supports the idea that nicotinic excitation encodes aversive valence (Fowler et al. 2011; Frahm et al. 2011; Wolfman et al. 2018). In addition, cholinergic projections from the MS to the MHb regulate the generalization of visually conditioned aversion, though this process involves M1 muscarinic receptors rather than nAChRs on MHb cholinergic neurons (Mu et al. 2022).

### 7.2 Anxiety

The MHb–IPN pathway also contributes to anxiety‐like behavior, with strong evidence implicating nicotinic signaling. MHb cholinergic neurons show higher activity in anxiogenic contexts, such as the open arms of the elevated plus maze (Sylwestrak et al. 2022). Accordingly, in most studies, activation of MHb–IPN transmission enhances anxiety‐like responses, such as avoidance and reduced exploration (Figure 4), in paradigms such as the elevated plus maze and forced swim test (Seigneur et al. 2018; Klenowski et al. 2023). Conversely, silencing this pathway reduces passive coping strategies and anxiety‐related responses (Figure 4) (Yamaguchi et al. 2013; Klenowski et al. 2023), with projections from the TS to the vMHb and central IPN implicated in these effects (Yamaguchi et al. 2013). However, opposite results have also been reported (Cho et al. 2020), notably in the dMHb‐lateral IPN pathway, which is substance‐Pergic rather than cholinergic (Handa et al. 2025; Wang et al. 2025). Additionally, dopamine release from VTA neurons onto the IPN may also participate in anxiety regulation (DeGroot et al. 2020). A subset of habenula‐IPN neurons expressing the transcription factor Otx2 appears particularly sensitive to chronic stress exposure during the peripubertal period, a developmental window that may confer increased vulnerability to anxiety‐related behaviors later in life (Rakotobe et al. 2024).

Nicotinic receptors are key mediators of anxiety encoding in this circuit. Blocking nAChRs in the IPN induces nicotine withdrawal–like symptoms in nicotine‐dependent mice, including anxiety (Zhao‐Shea et al. 2015). Intriguingly, deleting the β4 subunit has the opposite effect: it reduces sensitivity to anxiety paradigms and blunts aversive reactivity (Salas et al. 2003; Semenova et al. 2012; Husson et al. 2020). Conversely, boosting vMHb cholinergic signaling with a hypersensitive α4* nAChR increases anxiety (Pang et al. 2016). This paradox—where β4* nAChRs are required for withdrawal symptoms, yet their pharmacological inhibition alleviates anxiety and their activation exacerbates it—underscores the complex, bidirectional role of nicotinic signaling in the MHb‐IPN axis and highlights key gaps in our understanding of its regulation of anxiety.

### 7.3 Fear and Defensive Learning

IPN GABAergic neurons are also recruited by potential threats such as an approaching predator, and their silencing reduces defensive responses (Williams et al. 2025). Threat‐evoked responses decline with repeated exposure, suggesting that the IPN undergoes adaptive changes associated with defensive learning. Distinct subcircuits within the MHb–IPN pathway appear to contribute to different components of fear processing: IPN GABAergic neurons projecting to the LDTg are involved in threat learning, whereas IPN SST neurons mediate more generalized avoidance (Williams et al. 2025). In parallel, substance P‐expressing neurons in the dMHb, which project to the IPL, participate in contextual fear, and ablation of their afferents from the BAC impairs this function (Yamaguchi et al. 2013).

Synaptic mechanisms implicate nAChRs in fear modulation. Deleting or blocking CB1 receptors at MHb‐IPN synapses increases ACh transmission and reduces freezing responses in contextual fear conditioning experiments, while intra‐IPN infusion of the non‐specific nAChR antagonist mecamylamine—but not of glutamate receptor antagonists—in these mice restores aversive behavior (Soria‐Gómez et al. 2015). Conversely, inactivating GABA_B_ receptors at the MHb‐IPN synapses, or ablating MHb cholinergic neurons, suppresses neurotransmission and enhances the expression of aversive memories, and intra‐IPN infusion of ACh reduces fear memory expression (Zhang et al. 2016). This plasticity is restored during extinction (Koppensteiner et al. 2016). Together, these results show that increased IPN ACh levels facilitate fear extinction.

### 7.4 Depression and Stress Adaptation

The MHb–IPN pathway is also implicated in mood regulation and depressive‐like states, particularly anhedonia. In a model of stress‐enhanced sucrose seeking, optogenetic inhibition of IPN GABAergic neurons reduces stress‐induced motivation, while their photoactivation—or increased excitatory input from the MHb, potentially through nAChRs—potentiates reward‐seeking behavior (Klenowski et al. 2023). These neurons express SST and appear to gate a behavioral shift toward positive reinforcement under stress. Mice lacking the β4 subunit display reduced depression‐like behavior (Semenova et al. 2012), suggesting a role of MHb‐IPN nAChRs in these processes.

Consistent with this role in stress adaptation, alterations in the MHb–IPN axis have been observed in models of chronic stress and depression, and are associated with depression‐like behaviors, including anhedonia (Xu et al. 2018). At the molecular level, stress remodels the MHb transcriptome, modifying nAChR subunit expression (e.g., α5 and β4) and glutamate transporter levels (Yoo et al. 2022), likely altering MHb‐IPN excitability.

Moreover, this pathway interacts with the hypothalamic–pituitary–adrenal (HPA) axis, further reinforcing its involvement in the broader physiological stress response. Chemogenetic manipulation of MHb neurons alters corticosterone release after stress (Hsu et al. 2014), underscoring its function at the interface of emotional and neuroendocrine responses. Additionally, CRF release into the IPN contributes to nicotine withdrawal‐induced anxiety (Zhao‐Shea et al. 2015). As the MHb‐IPN axis is heavily cholinergic and nAChR‐driven, these findings support a broader nicotinic control over neuroendocrine stress physiology.

## 8 Role of Endogenous Nicotinic Modulation in Social Interactions

The MHb–IPN pathway is emerging as a key regulator of social affective behaviors by integrating cholinergic, glutamatergic, and dopaminergic signals to shape processes such as social interaction, conflict resolution and motivation (Ables et al. 2023).

In mice, recent studies have provided direct evidence linking this pathway to social novelty exploration. IPN neuronal activity increases during social interactions, yet only after repeated social exposure, as the stimuli become familiar (Molas, Zhao‐Shea, et al. 2017). Optogenetic manipulation of IPN neurons bidirectionally shifts novelty preference (Figure 4), suggesting that the IPN modulates the motivational salience of familiar cues. Dopamine signals within the IPN increase during novel social interaction but not during familiar encounters, implicating the VTA–IPN circuit, and particularly its projections to the LDTg, in motivated exploratory behavior (Molas et al. 2024). Furthermore, the IPN–LDTg projection contributes to the processing of novel social olfactory cues during nicotine withdrawal (Monical and Mcgehee 2025), reinforcing the idea that this circuit integrates motivational and affective information to regulate social behavior. Mice lacking the β4 subunit exhibit altered interactions with familiar conspecifics (Salas et al. 2013) and enhanced novelty‐induced activity (Husson et al. 2020), suggesting that nicotinic signaling contributes to social engagement and salience attribution.

Beyond novelty, the MHb–IPN circuit appears to regulate affiliative and hierarchical social behaviors. In zebrafish, the dorsal habenula (homologous to rodent MHb) modulates social defeat and conflict resolution via α7 nAChR activation (Kinoshita and Okamoto 2023). Moreover, two subregions of the dorsal habenula exert opposite effects on social outcome: silencing either side biases the fish toward winning or losing in social conflict (Chou et al. 2016). These findings suggest a finely tuned control of social strategy via habenular outputs. In mice, similar principles apply. Postnatal ablation of MHb neurons markedly reduces ACh levels in the IPN and produces behavioral impairments including hyperactivity, impulsivity, maladaptation, and cognitive inflexibility (Kobayashi et al. 2013). Optogenetic activation of the MHb–IPN cholinergic pathway promotes social submission by inhibiting serotonergic neurons in the MnR, whereas disrupting cholinergic transmission enhances dominance behavior (Matsumata et al. 2025).

Together, these findings identify the MHb–IPN pathway as a conserved circuit for regulating social behaviors, from novelty detection to social conflict resolution. Nicotinic signaling within this circuit may serve as a crucial modulator of social motivation, behavioral flexibility, and hierarchy‐related responses.

## 9 Future Directions and Open Questions

Despite major advances, fundamental questions remain about the nicotinic modulation of the MHb–IPN pathway. While the anatomical and functional complexity of this circuit is increasingly recognized, we are only beginning to resolve how nAChR subunit composition and function vary across specific neuronal populations—and how these properties change with context, physiological state, or experience. The MHb–IPN pathway expresses one of the densest and most diverse arrays of nAChRs in the brain, yet the precise localization of these subtypes, their cell‐specific functional roles, and their regulation under physiological and pathological conditions remain incompletely understood. Although α3β4* and α5* nAChRs have been clearly implicated in nicotine aversion, the roles of less‐characterized subunits such as α2 or β3 remain largely undefined. Moreover, the endogenous source of ACh within the MHb is still unclear, particularly in light of the presence of functional nAChRs on MHb somata and axons in the absence of known cholinergic inputs (Passlick et al. 2018). Recent findings of feedforward cholinergic excitation suggest a form of intra‐habenular transmission (Chung et al. 2023), but direct evidence for this mechanism remains sparse. Addressing these knowledge gaps will require both anatomical and functional refinement. Mapping receptor subunit localization at single‐cell resolution using spatial transcriptomics and multiplexed in situ hybridization should be complemented by functional profiling of receptor subtypes in genetically defined neuronal populations.

A major open question concerns how distinct IPN output pathways mediate diverse behavioral outcomes. The IPN projects to several neuromodulatory and behavioral control centers, including the LDTg, raphe nuclei, and NI, each implicated in specific processes. Notably, IPN‐LDTg projections have been linked to nicotine aversion (Wolfman et al. 2018), reward (Jehl et al. 2025), and withdrawal symptoms (Monical and Mcgehee 2025), as well as to anxiety and coping (Klenowski et al. 2023), fear learning (Williams et al. 2025), and social novelty preference (Molas et al. 2024). Determining how these parallel subcircuits are organized, and how their nicotinic modulation shapes target activity, will be critical to understanding how the IPN gates different motivational and emotional states. Combining cell‐type–resolved tracing, functional imaging, and projection‐specific manipulations will be essential to map how nicotinic signaling influences information flow from the IPN to its downstream targets.

Addressing these questions will benefit from newly developed methods that allow precise manipulation of nAChRs with unprecedented pharmacological and spatial control (Kramer et al. 2013; Mondoloni et al. 2019; Paoletti et al. 2019; Colleoni et al. 2025). Caged nicotinic agonists (Banala et al. 2018, 2024; Passlick et al. 2018) and antagonists—particularly those sensitive to two‐photon excitation (Warther et al. 2010)—allow subcellular mapping of functional nAChRs in situ. When applied in behaving animals (Paoletti et al. 2019; Durand‐de Cuttoli et al. 2020; Ma et al. 2023; McClain et al. 2023), these photochemical tools can bridge receptor‐level activity to circuit function and behavior. Complementary chemogenetic strategies permit manipulation of nAChRs with both cell‐type and subunit specificity. These include hypersensitive nAChRs carrying single‐point mutations in the channel pore that increase agonist sensitivity by over 100‐fold, enabling selective activation of α4* or α6* nAChRs in knock‐in mice (Tapper et al. 2004; Drenan et al. 2008; Fonck et al. 2009). In parallel, cysteine‐substituted subunits permit covalent tethering of agonists or antagonists—some of which are photoresponsive—near the ACh‐binding site, allowing for either rapid and reversible receptor manipulation (Tochitsky et al. 2012; Durand‐de Cuttoli et al. 2018) or sustained antagonism in darkness (Jehl et al. 2025). These engineered subunits can be delivered virally to specific populations (Durand‐de Cuttoli et al. 2018; Mondoloni et al. 2019), or expressed from knock‐in alleles (e.g., β2* and β4*) to enable endogenous targeting (Jehl et al. 2025). Collectively, these tools offer unprecedented temporal, spatial, and molecular resolution for dissecting nAChR function in vivo. Finally, the recent development of optical ACh sensors (Jing et al. 2018, 2020; Mineur and Picciotto 2023; Favier et al. 2024) provides the means to monitor real‐time fluctuations in cholinergic signaling during behavior or pharmacological manipulation, a critical step for understanding endogenous ACh dynamics across MHb‐IPN subregions and behavioral states.

Beyond basic neuroscience, this pathway holds significant therapeutic potential. The α5, α3, and β4 nAChR subunits regulate both the rewarding and aversive properties of nicotine (Wills et al. 2022; Jehl et al. 2025), positioning the MHb–IPN axis as a critical regulator of nicotine intake. Strategies targeting these subtypes—whether via selective agonists, antagonists, or allosteric modulators—could inspire new smoking cessation therapies that reduce craving while preserving or enhancing aversive responses to the drug. Moreover, given the emerging roles of the MHb–IPN circuit in anxiety, depression, and social behavior regulation, modulating its nicotinic tone may also be beneficial in affective and neuropsychiatric disorders, particularly in individuals carrying polymorphisms in nicotinic receptor genes.

Importantly, the MHb–IPN pathway may serve as a shared substrate for multiple psychiatric conditions that frequently co‐occur with nicotine dependence. For instance, smoking and opioid use disorders exhibit strong comorbidity (Rajabi et al. 2019), potentially reflecting the high density of MORs in this circuit and their ability to modulate MHb‐IPN transmission. Similarly, the elevated prevalence of smoking in individuals with depression and generalized anxiety disorder (Molas, DeGroot, et al. 2017) aligns with the MHb‐IPN pathway's well‐documented role in anxiety and aversion encoding. These links underscore the broader therapeutic potential of targeting nicotinic signaling in this pathway—not only for treating nicotine addiction but also for addressing a spectrum of comorbid psychiatric conditions.

In summary, a deeper understanding of the cell‐type–specific roles of nAChRs in the MHb–IPN axis will be essential to elucidate how this circuit processes aversive and emotional states, and how its dysfunction contributes to psychiatric and substance use disorders. Continued integration of transcriptomic, optical, and behavioral methods will be essential to fully unravel the complexity—and therapeutic potential—of this ancient cholinergic circuit.

## Author Contributions

**Maria Ciscato:** writing – original draft, writing – review and editing. **Mathilde Chouvaeff:** writing – original draft, writing – review and editing. **Alexandre Mourot:** supervision, visualization, writing – original draft, writing – review and editing.

## Funding

This work was supported by the Agence Nationale de la Recherche (ANR‐10‐IDEX‐0001 PSL‐Neuro, ANR‐21‐CE16‐0012 CHOLHAB, ANR‐21‐CE37‐0026 NICOPTOTOUCH), the Human Frontier Science Program (RGP0035/2020), the Fondation pour la Recherche Médicale (EQU201903007961, FDT202404018105), and the National Institutes of Health (NS111600).

## Conflicts of Interest

The authors declare no conflicts of interest.

## Data Availability

The authors have nothing to report.
